# Relaxin abrogates renal interstitial fibrosis by regulating macrophage polarization via inhibition of Toll-like receptor 4 signaling

**DOI:** 10.18632/oncotarget.15483

**Published:** 2017-02-18

**Authors:** Lei Chen, Ming-Lei Sha, Deng Li, Yi-Ping Zhu, Xing-Jie Wang, Chen-Yi Jiang, Shu-Jie Xia, Yi Shao

**Affiliations:** ^1^ Department of Urology, Shanghai General Hospital, Shanghai Jiao Tong University School of Medicine, Shanghai, China; ^2^ Department of Geriatric, Shanghai General Hospital, Shanghai Jiao Tong University School of Medicine, Shanghai, China

**Keywords:** renal fibrosis, relaxin, Toll-like receptor 4, macrophage polarization

## Abstract

Renal fibrosis is a common feature of chronic kidney disease (CKD). To inhibit the CKD process, it is important to prevent renal fibrosis, though CKD remains incurable. Renal fibrosis can be inhibited by relaxin in several experimental models, but the mechanism of relaxin for antifibrotic potential is still not clear. And here we have studied the role of relaxin in macrophage polarization and renal inflammation after unilateral ureteral obstruction (UUO). Our results show that relaxin can downregulate the Toll-like receptor (TLR) 4 signaling, shift macrophage polarization toward the M2 phenotype and ameliorat renal fibrosis in the early stages of UUO. *In vitro* experiments, it has been confirmed that relaxin can downregulate the TLR4 signaling and induce the M2 macrophage transition. Furthermore, the transitional actions of macrophage phenotype induced by relaxin are significantly blocked by TAK-242, a TLR4 antagonist, *in vitro* experiments. Thus, there is a novel mechanism of relaxin for antifibrosis that shifts macrophage polarization toward the M2 phenotype via inhibition of TLR4 signaling.

## INTRODUCTION

Chronic kidney disease (CKD), regardless of the initial cause of the renal disease, is characterized by the progressive deterioration of kidney function, the relentless accumulation and deposition of extracellular matrix, and progressive tissue fibrosis. The incidence and prevalence of CKD has been estimated to be 8–16% worldwide and is increasing [1]. Renal fibrosis is a common feature of CKD, contributes prominently to the progressive loss of organ structure and function and leads to end-stage renal failure [2]. To inhibit the CKD process, it is important to prevent renal fibrosis, though CKD remains incurable. Macrophages (MΦ), which have diverse functions and phenotypic plasticity, have been recognized as key factors in renal fibrosis. MΦ have been classified into classically activated M1 and alternatively activated M2 subgroups. M1 macrophages play a pro-inflammatory role, enhancing renal inflammation by secreting pathogenic mediators, resulting in renal fibrosis. M2 macrophages play an anti-inflammatory role, suppressing renal inflammation by releasing anti-inflammatory mediators, such as interleukin (IL)-10, resulting in reduced renal fibrosis [3, 4, 5].

Relaxin is an ovarian and cardiovascular hormone that has emerged as a rapid-acting but safe antifibrotic that inhibits renal fibrosis in several experimental models, including the unilateral ureteral obstruction (UUO) and renal ischemia/reperfusion injury models [6, 7, 8]. The clinical assessment of the vasodilatory benefits in acute heart failure has been well developed, but the mechanism of relaxin for the antifibrotic potential is not clear [9].

After UUO or renal ischemia/reperfusion injury, changing the macrophage polarization conditions plays an important role in renal fibrosis, but whether relaxin ameliorates renal fibrosis by changing the macrophage polarization is still unknown. However, relaxin has the potential to shift macrophage polarization toward the M2 phenotype. Figueiredo et al. found that in response to inflammatory stimuli, relaxin can suppress expression of the typical M1-cytokine IL-1β in macrophages, thus promoting the acquisition of an immunosuppressive M2 phenotype in macrophages [10].

Toll-like receptors (TLRs) are an innate family of receptors that can sense tissue damage and orchestrate a cascade of inflammation. Recent evidence indicates that TLR4-mediated inflammation is a critical pathogenic link between innate immunity and renal fibrosis. TLR4, which is upregulated in mouse kidneys after UUO, can also promote fibrosis after UUO [11]. TLR4 also plays an important role in the transduction of polarization phenotype response and the release of pro-inflammatory cytokines in macrophages. HMGB1, the ligand of TLR4, and TLR4 signaling pathway expression are essential for the M1 macrophage transition [12, 13]. Otherwise, global TLR4 deficiency shifts macrophage polarization toward the M2 phenotype and consequently reduces adipose tissue inflammation [14]. However, it is unclear whether relaxin ameliorates renal fibrosis by inducing the macrophage phenotype transition. We hypothesized that relaxin induces M2 macrophage polarization by inhibiting the TLR4-NF-κB signaling pathway and alleviates the inflammation and renal fibrosis at the early stages of UUO. Our results showed that relaxin can downregulate the TLR4-NF-κB signaling pathway, shift macrophage polarization toward the M2 phenotype and ameliorate renal fibrosis at the early stages of UUO. *In vitro* experiments confirmed that relaxin can downregulate the TLR4-NF-κB signaling pathway and induce the M2 macrophage transition. Furthermore, the macrophage phenotype transition actions of relaxin were significantly blocked by TAK-242, a TLR4 antagonist, *in vitro*.

## RESULTS

### Relaxin alleviates fibrosis following UUO

**Figure 1:**
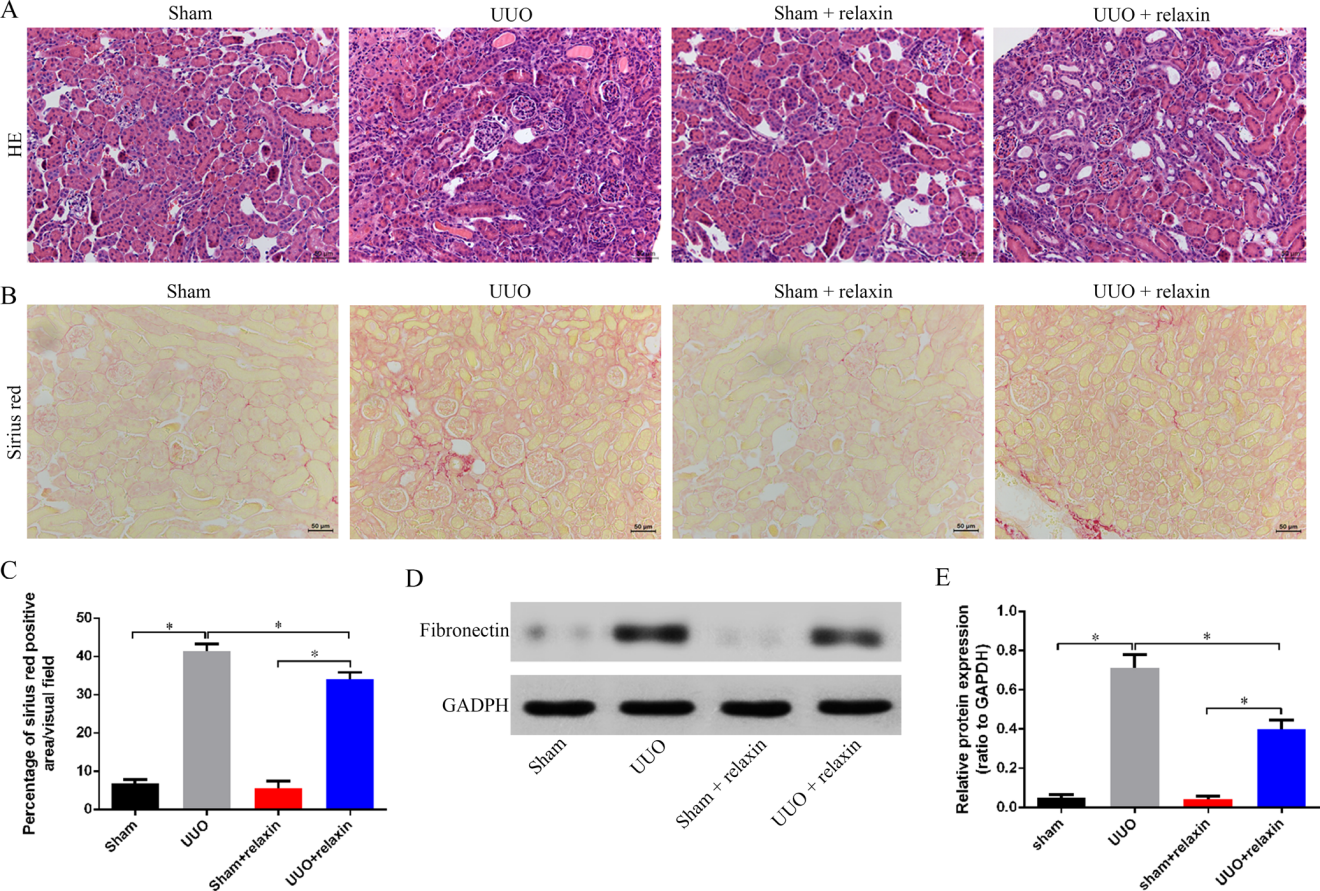
Relaxin alleviates fibrosis following UUO (**A**–**B**) Representative images (eight visual fields for each tissue analyzed) of H&E (A), Sirus red (B) staining of kindrys frong the indicated experimental groups. Scale bars, 50 μm. (**C**) Interstitial fibrosis on basis of Sirius red staining. *n* = 8 per group;*p < 0.05. (**D**, **E**) Whole kidney lysates from kidneys of the mice pretreated with deionized water or relaxin following UUO (sham, UUO, sham + relaxin and UUO + relaxin) were analyzed for changes in fibrosis (fibronectin) by western blot analysis. Expression of the indicated proteins in the kidneys was analyzed by densitometry normalized to glyceraldehyde 3-phosphate dehydrogenase (GADPH) and expressed as mean ± sd. *n* = 8 per group; *p < 0.05.

To determine the functional significance of relaxin in renal fibrosis, we analyzed fibrotic kidneys following hematoxylin and eosin (H&E), Sirius red and protein fibronectin. Mice were pretreated with relaxin (0.5 mg/kg per day) or deionized water as control. Their kidneys were assessed at day 5 (in the early stage of UUO and when renal fibrosis is well established). H&E and Sirius red staining showed that marked interstitial inflammation and fibrosis occurred 5 days after UUO and that relaxin can reduce cell infiltration and interstitial fibrosis (Figure 1A–1C). Compared with the kidneys of mice pretreated with relaxin, the kidneys of day 5 UUO-injured mice had higher fibronectin levels (Figure 1D, 1E), suggesting that relaxin can alleviate renal fibrosis after UUO.

### Relaxin shifts macrophage polarization toward the M2 phenotype *in vivo* following UUO

**Figure 2:**
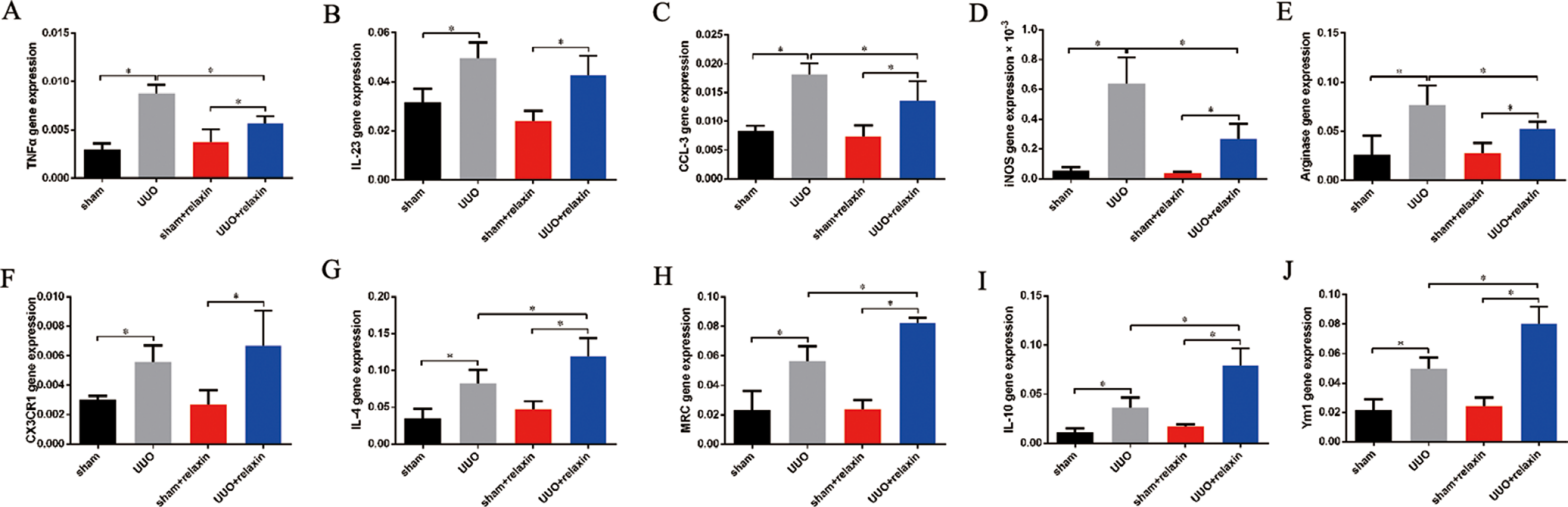
Relaxin shifts macrophage polarization toward the M2 phenotype *in vivo* following UUO (**A**–**J**) Gene expression analysis of M1 markers, including tumor necrosis factor-α (TNF-α), interleukin (IL)-23, chemokine (C-C motif) ligand 3 (CCL3), inducible nitric oxide synthase (iNOS), and M2 markers, including arginase, CX3CR1, IL-4, mannose receptor (MRC), IL-10, Ym1 on macrophages isolated from kidneys of the mice pretreated with deionized water or relaxin following UUO. Experiments were performed at least three times and gene expression data were normalized to GADPH, analyzed, and represented as mean ± sd; *n* = 8 per group; *p < 0.05.

To characterize the differential phenotypes of macrophages 5 days following UUO, macrophages were isolated from the obstructed and contralateral kidneys and analyzed for the expression of M1 and M2 markers by real-time PCR. Five days following UUO, gene expression analysis of macrophages from the kidneys revealed increased both M1 and M2 markers (Figure 2A–2J). Relaxin pretreatment caused significant increases in the gene expression levels of most renal macrophage M2 markers (MRC (mannose receptor), IL-4, arginase, IL-10, and Ym1); only the CX3CR1 (M2) gene was not differentially expressed in the macrophages from the two groups (Figure 2E–2J). However, the macrophage M1 markers (iNOS (inducible nitric oxide synthase), TNF-α (tumor necrosis factor-α), CCL-3 (chemokine (C-C motif) ligand 3), IL-23) were significantly decreased after relaxin pretreatment (Figure 2A–2D).

### Relaxin shifts macrophage polarization toward the M2 phenotype *in vitro*

**Figure 3:**
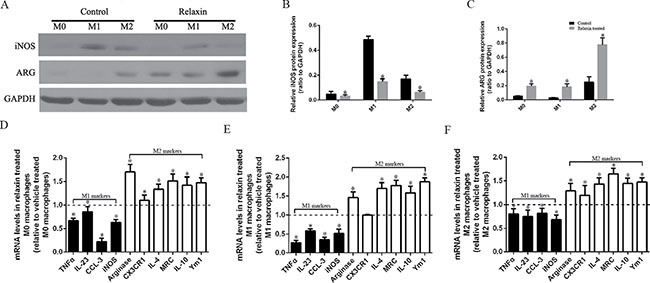
Relaxin shifts macrophage polarization toward the M2 phenotype *in vitro* Raw 264.7 cells were treated with deionized water (M0), interferonγ(IFNγ) (M1) or IL-4(M2) and (**A**–**C**) analyzed for the expression of M1 marker iNOS and M2 marker arginase (Arg) after treated with vehicle (deionized water) or relaxin by western blot analysis. Protein expression data were normalized to GADPH, analyzed, and represented as mean ± sd; *p < 0.05 vs. vehicle treated groups(control groups). (**D**–**F**) M0, M1 and M2 macrophages were analyzed for the expression of M1 (TNF-α, IL-23, CCL3, iNOS) and M2 (arginase, CX3CR1, IL-4, MRC, IL-10, Ym-1) genes after treated with vehicle (deionized water) or relaxin by real-time PCR. Experiments were performed at least three independent times and gene expression data were normalized to GAPDH, analyzed, and represented as mean ± sd; *p < 0.05 vs. vehicle treated groups (control groups).

To further investigate the correlation between relaxin treatment and macrophage polarization, we polarized Raw264.7 cells to M1 or M2 macrophages *in vitro* using interferon-γ (IFNγ) or IL-4 and treated them with relaxin or deionized water as vehicle control, respectively. After treatment with relaxin, iNOS protein expression was decreased and Arg expression was increased in M0, M1 and M2 macrophages (Figure 3A–3C). The expression levels of M1 marker genes (iNOS, TNF-α, CCL-3, IL-23) were upregulated and M2 marker genes (arginase, CX3CR1, IL-4, Arg-1, IL-10, Ym1) were downregulated after treatment with relaxin in Raw264.7 cells, corroborating our *in vivo* data showing that relaxin treatment can increase the propensity to polarize M0 macrophages to the M2 phenotype (Figure 3D). Similarly, when M1 macrophages or M2 macrophages were treated with relaxin, the expression levels of most of the M1 marker genes decreased while M2 markers increased (Figure 3E, 3F), indicating that relaxin can shift M1 macrophage polarization toward the M2 phenotype.

### Relaxin downregulates the TLR4-NF-κB signaling pathway *in vivo* following UUO

**Figure 4:**
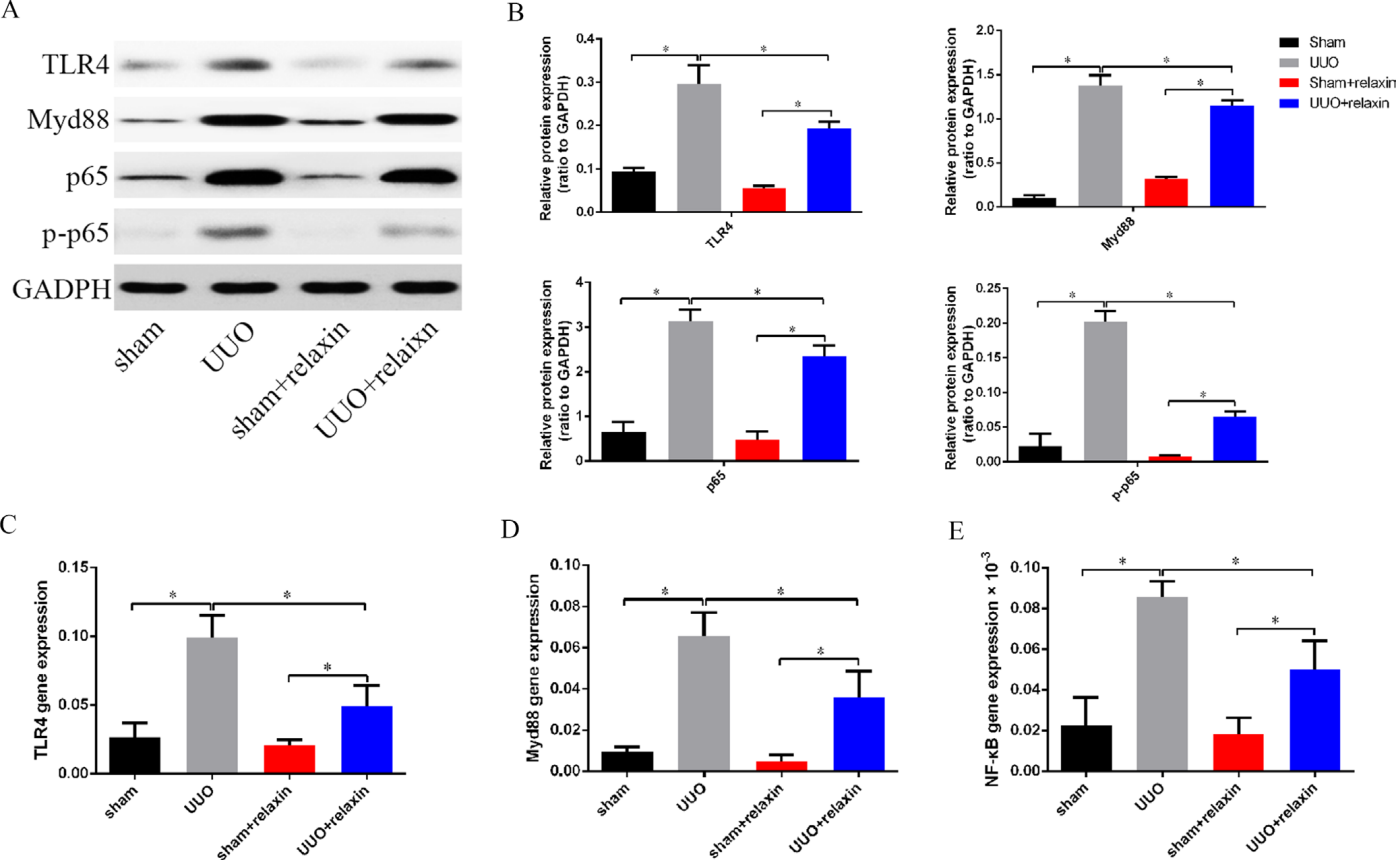
Relaxin downregulates the TLR4-NF-κB signaling pathway *in vivo* following UUO (**A**, **B**) Whole kidney lysates from kidneys of the mice pretreated with deionized water or relaxin following UUO (sham, UUO, sham + relaxin and UUO + relaxin) were analyzed for changes in TLR4, Myd88, p65, p-p65 by western blot analysis. Expression of the indicated proteins in the kidneys was analyzed by densitometry normalized to GADPH and expressed as mean ± sd; *n* = 8 per group; *p < 0.05. (**C**–**E**) Macrophages isolated from kidneys of the mice pretreated with deionized water or relaxin following UUO were analyzed for the expression of TLR4, Myd88 and p65 genes. Experiments were performed at least three independent times and gene expression data were normalized to GAPDH, analyzed, and represented as mean ± sd; *n* = 8 per group;*p < 0.05.

Given the significance of the TLR4 signaling pathway for M1 macrophage transition, we hypothesized that relaxin induces M2 macrophage polarization by inhibiting the TLR4-NF-κB signaling pathway. The mice were pretreated with relaxin or deionized water as control, and the kidneys were collected at day 5 following UUO. TLR4, Myd88, NF-κB (p65) and phospho-NF-κB (p-p65) were measured by western blotting and real-time PCR. TLR4, Myd88, p65 and pp65 expression levels were upregulated at day 5 following UUO, while relaxin significantly decreased their mRNA and protein expression levels (Figure 4A–4E).

### Relaxin downregulates the TLR4-NF-κB signaling pathway *in vitro*

**Figure 5:**
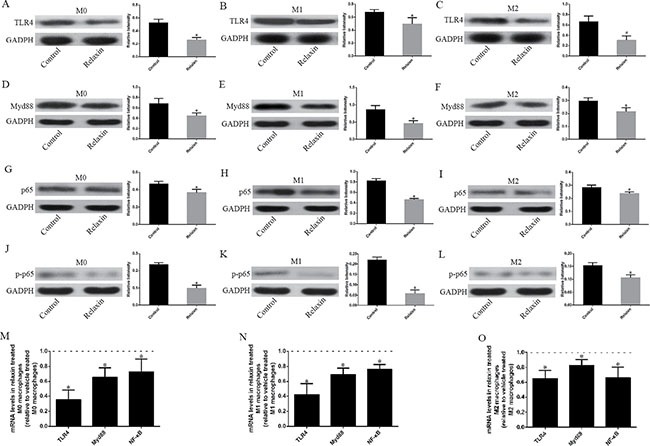
Relaxin downregulates the TLR4-NF-κB signaling pathway *in vitro* Raw 264.7 cells were treated with deionized water (M0), interferonγ (IFNγ) (M1) or IL-4(M2) and (**A**–**L**) analyzed for the expression of TLR4, Myd88, p65 after treated with vehicle (deionized water) or relaxin by western blot analysis. Protein expression data were normalized to GADPH, analyzed, and represented as mean ± sd; *p < 0.05 vs. vehicle treated groups (control groups). (**M**, **N**, **O**) M0, M1 and M2 macrophages were analyzed for the expression of TLR4, Myd88, p65, p-p65 genes after treated with vehicle (deionized water) or relaxin by real-time PCR. Experiments were performed at least three independent times and gene expression data were normalized to GAPDH, analyzed, and represented as mean ± sd; *p < 0.05 vs. vehicle treated groups (control groups).

To elucidate the significance of relaxin in the regulation of the TLR4 signaling pathway of the different macrophage phenotypes *in vitro*, and as described earlier in this study, we polarized Raw264.7 cells to M1 or M2 macrophages and analyzed the mRNA and protein expression profiles of the TLR4 signaling pathway of the different macrophage phenotypes after treatment with relaxin or vehicle. Compared with those treated with vehicle, the mice treated with relaxin downregulated TLR4, Myd88, p65 and pp65 protein expression in both M0, M1 and M2 macrophages (Figure 5A–5L). Consistent with the protein expression data, real-time PCR results also indicated that relaxin could inhibit the TLR4 signaling pathway. TLR4, Myd88, p65 and pp65 genes were expressed at low levels after relaxin treatment in the different macrophage phenotypes compared with those treated with vehicle (Figure 5M–5O).

### The effects of relaxin on macrophage polarization regulation are blocked by the TLR4 antagonist TAK-242 *in vitro*

**Figure 6:**
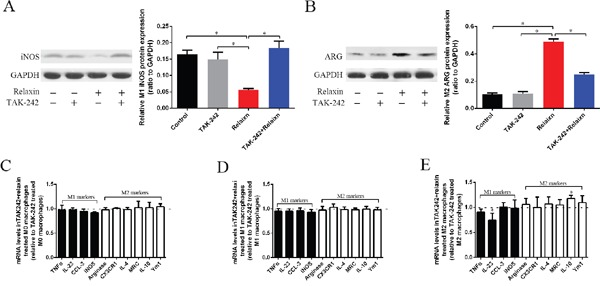
The effects of relaxin on macrophages polarization regulation are blocked by the TLR4 antagonist TAK-242 *in vitro* (**A**, **B**) Raw 264.7 cells were treated with IFNγ (M1) to induce the macrophage to M1 macrophages, the cells were pre-treated with TAK-242 or vehicle (deionized water) for 1 h and were continuously treated with vehicle (deionized water) or relaxin for over 72 h and analyzed for the expression of M1 marker iNOS (**A**) and M2 marker arginase (Arg) (**B**) by western blot analysis. Protein expression data were normalized to GADPH, analyzed, and represented as mean ± sd; *p < 0.05. (**C**–**E**) M0, M1 and M2 macrophages were analyzed for the expression of M1 (TNF-α, IL-23, CCL3, iNOS) and M2 (arginase, CX3CR1, IL-4, MRC, IL-10, Ym-1) genes after treated with relaxin and TAK-242 or TAK-242 only by real-time PCR. Experiments were performed at least three independent times and gene expression data were normalized to GAPDH, analyzed, and represented as mean ± sd; *p < 0.05 vs. treated with TAK-242 only.

After inducing Raw264.7 cells into M1 or M2 macrophages, the cells were pre-treated with TAK-242 (1 μM) or deionized water as vehicle control for 1 h and were continuously treated with relaxin (100 ng/ml; 16.8 nmol/l) for 72 h. Untreated cells and cells treated with TAK-242 alone (1 μM) for 72 h were used as appropriate controls. Treatment of different phenotypes of macrophages with relaxin can shift macrophage polarization toward the M2 phenotype *in vitro* and *in vivo* (Figures 2A–2J, 3A–3F). However, compared with their expression in the cells treated with TAK-242 only, the M1 markers (TNF-α, IL-23, CCL-3, iNOS,) and most of the M2 markers (arginase, CX3CR1, IL-4,MRC, Ym1) were not differentially expressed in the M0, M1 and M2 macrophages treated with TAK-242 and relaxin; only the IL-10 (M2) genes were increased in the M2 macrophages after treated with both TAK-242 and relaxin (Figure 6C–6E). Consistent with the gene expression data, the protein expression profiles of iNOS (M1) in M1 macrophage and Arg (M2) in M2 macrophage were not different between those treated with TAK-242 and those treated with TAK-242 and relaxin (Figure 6A, 6B). The results indicated that the effects of relaxin on regulating macrophage polarization are blocked by the TLR4 antagonist TAK-242 *in vitro*.

## DISCUSSION

This study explored whether the H2 relaxin ameliorated renal fibrosis at the early stage of UUO by shifting macrophage polarization toward the M2 phenotype. Consistent with previous studies, relaxin can promote the acquisition of an immunosuppressive M2 phenotype in macrophages; M2 macrophages suppress kidney inflammation by releasing anti-inflammatory mediators, such as IL-10, thus inhibiting renal fibrosis [3, 4, 5, 10]. We also found that relaxin can downregulate the TLR4-NF-ΚB signaling pathway at all levels. Surprisingly, however, the macrophage phenotype transition function of relaxin was significantly blocked by the TLR4 antagonist TAK-242 *in vitro*. Therefore, we demonstrated for the first time that H2 relaxin can shift macrophage polarization toward the M2 phenotype by downregulating the TLR4-NF-ΚB signaling pathway, thus alleviating renal fibrosis.

Relaxin is a fast-acting but safe antifibrotic that inhibits renal fibrosis in UUO [6, 7, 8]. Recent studies have demonstrated that relaxin abrogates the TGF-β1/pSmad2 axis through an RXFP1-pERK1/2-nNOSNO-cGMP-dependent pathway and regulates the collagen-degrading matrix-degrading matrix metalloproteinases (MMPs) (MMP-1/−13, MMP-2 and MMP-9) [15, 16, 17]. However, our results show that relaxin can shift macrophage polarization toward the anti-inflammatory macrophage phenotype (M2), thus ameliorating renal fibrosis at the early stage of UUO. Our results indicate that relaxin can suppress fibrosis indirectly through anti-inflammatory actions. Consistent with our result, recent studies have shown that relaxin has the potential to shift macrophage polarization toward the M2 phenotype. Figueiredo et al. found that in response to inflammatory stimuli, relaxin can suppress expression of the typical M1-cytokine IL-1β in macrophages, thus promoting acquisition of an immunosuppressive M2 phenotype in macrophages [10]. Binder et al. investigated the mechanism of relaxin-enhanced breast tumor growth and found that tumor growth was enhanced because relaxin can switch macrophages toward the M2 phenotype [18].

MΦ show a phase-dependent balance of pro-inflammatory and anti-inflammatory effects in UUO. In the early stages of UUO, the pro-inflammatory macrophage (M1) is the predominant macrophage phenotype, directly inducing renal fibrosis by releasing pathogenic mediators such as TNF-α, IL-1β, CCL2 and reactive oxygen species (ROS). In the late stage of disease, MΦ undergo a switch to an anti-inflammatory macrophage (M2), which can suppress renal inflammation by secreting IL-10, leading to reduced renal fibrosis [4]. Consistent with previous studies, our findings indicate that relaxin could switch M1 macrophages to an M2 phenotype at the early stage of obstructive injury, resulting in reduced renal fibrosis.

TLRs, activated by pathogen-associated motifs and the release of endogenous stress ligands, can orchestrate an inflammatory response during tissue injury. TLR4, expressed in a wide range of cell types, including macrophage and renal epithelial cells, provides an important response after UUO [19]. TLR4 expression is significantly increased after UUO and induces renal fibrosis [20]. One day after unilateral ureteral obstruction, TLR4-deficient mice had more tubular damage and fewer proliferating tubular epithelial cells than WT mice but developed considerably less renal fibrosis [11]. Their studies suggest that UUO can induce a dramatic increase in TLR4 expression, therefore promoting renal fibrosis. In our study, we found that relaxin can downregulate the TLR4-NF-ΚB signaling pathway, indicating that relaxin can indirectly suppress fibrosis through anti-inflammatory activity.

Considering our finding that relaxin could switch M1 macrophages to an M2 phenotype and that the mechanism by which relaxin modulates macrophage polarization and function is unknown, we investigated whether relaxin shift macrophage polarization toward the M2 phenotype via inhibition of TLR4 signaling. We found that the macrophage phenotype transition action of relaxin was significantly blocked by the TLR4 antagonist TAK-242 *in vitro*. This finding indicates that relaxin shift macrophage polarization toward the M2 phenotype via inhibition of TLR4 signaling. Consistent with our results, previous studies have suggested that TLR4 also plays an important role in the transduction of polarization phenotype signals and the release of pro-inflammatory cytokines in macrophages. Delta-like 4 (DLL4) can increase M1 macrophage markers, such as IL-12 and iNOS expression, and can reprogram macrophages to the M1 phenotype via the TLR4/NF-κB-dependent pathway [21]. HMGB1, the ligand of TLR4, can promote renal fibrosis by facilitating the M1 macrophage phenotype [13]. Zhou et al. also found that curcumin can inhibit M1 macrophage polarization via the inhibition of expression along the TLR4 signaling pathway [12]. The mechanisms of macrophage heterogeneity remain unknown; however, it is reasonable to confirm that the TLR4 signaling pathway is a candidate switch between M1 and M2 phenotypes.

This study has several inherent limitations. First, we used Raw264.7 cells rather than other macrophages to investigate the effect of relaxin on macrophage polarization via TLR4 signaling pathways *in vitro*. Further studies should investigate relaxin-mediated macrophage polarization in macrophages isolated from mice following UUO and other macrophages. Additionally, it is necessary to use *in vivo* transgenic mouse experiments to further validate the effect of relaxin-mediated macrophage polarization and its mechanism.

Taken together, these results have demonstrated a novel mechanism by which relaxin ameliorates renal fibrosis by regulating macrophage polarization. The TLR4 signaling pathway is critically required for relaxin to shift macrophage polarization toward the M2 phenotype *in vitro*. This essential requirement is achieved through downregulation of the TLR4-NF-ΚB signaling pathway. However, the observation that TLR4 expression is low under normal physiological conditions and that it is upregulated in models of UUO may explain why the antifibrotic effects of relaxin are only observed under pathological conditions.

## MATERIALS AND METHODS

### Animal model

C57BL/6 male mice were supplied by the laboratory animal center of our institute. Ethical approval for all animal experiments was obtained from the Institutional Animal Care and Use Committee at Shanghai First People's Hospital of Shanghai Jiao Tong University.

Mice were preventatively treated with recombinant H2 relaxin (PeproTech, Rocky Hill, NJ, USA) (0.5 mg/kg per day) or deionized water (PeproTech, Rocky Hill, NJ, USA) as control via subcutaneously implanted osmotic minipumps (model 1007D; Alzet, Cupertino, CA, USA) from 2 days before UUO until 5 days after injury. In various models of renal disease, regardless of etiology, this dose of relaxin had previously been used to successfully prevent or reverse fibrosis progression [7, 8].

As described in a previous study, a mouse model of UUO was used as an experimental model of primary tubulointerstitial fibrosis [22]. After general anesthesia, mice underwent ligation of the right ureter, with the left kidney remaining intact. The right ureter was exposed after a midline abdominal incision. The ligated animals were completely obstructed 1 cm below the renal pelvis with 5.0 silk ligature; sham operated animals were manipulated similarly but not ligated. Five days after surgery, the kidneys were collected, rinsed with isotonic saline, dissected and stored in liquid nitrogen or fixed in 4% formaldehyde for further analysis.

### Cell culture and differentiation

Raw264.7 macrophages were obtained from the Shanghai cell bank of the Chinese Academy of Sciences. Raw264.7 macrophages were cultured in Dulbecco's modified Eagle's medium (DMEM) supplemented with 10% fetal bovine serum (FBS) and 1% penicillin/streptomycin (Life Technologies, Waltham, MA, USA). The cells, induced to differentiate as previously described, were stimulated with 100 U/ml murine recombinant IFNγ (PeproTech, Rocky Hill, NJ, USA) or 20 ng/ml murine recombinant IL-4 (PeproTech, Rocky Hill, NJ, USA) for 24 h to induce M1 or M2 polarization, respectively [23].

After Raw264.7 cells were induced into M1 or M2 macrophages, the cells were pre-treated with TAK-242 (1 μM; MedChemExpress, Monmouth Junction, NJ, USA) or deionized water (PeproTech, Rocky Hill, NJ, USA) as vehicle control for 1 h and were continuously treated with relaxin (100 ng/ml; 16.8 nmol/l) for 72 h. Cells treated with deionized water or TAK-242 alone (1 μM) for 72 h were used as appropriate controls.

### Histological analysis

Kidney tissues were embedded in paraffin after fixation in 4% paraformaldehyde (PFA) in phosphate-buffered saline (PBS) for 24 h. A paraffin microtome with stainless steel knives was used to cut the paraffin blocks into 8-μm sections. Before immunostaining, the renal tissue sections were mounted on glass slides, dewaxed in xylene, rehydrated with decreasing concentrations of ethanol, washed in PBS, and then stained with H&E. After staining, the sections were dehydrated with increasing concentrations of ethanol and xylene.

To evaluate collagen deposition, the renal tissue sections were stained with Sirius red as previously described [23]. The renal tissue sections were deparaffinized with xylene and then rehydrated in water with graded ethanol washes. After two washes in acidified water, the sections were incubated in Picrosirius red for 1 h. The sections were then dehydrated, cleared, and mounted in a resinous medium. The area of collagen deposition was measured using color image analysis software (Image-Pro Plus, Media Cybernetics, Houston, TX, USA).

### Isolation and enrichment of renal macrophages

CD11b-expressing cells in the kidney single-cell suspensions were enriched using mouse CD11b Microbeads and MACS columns (Milteni Biotec Auburn, CA, USA) following the manufacturer's protocol.

### Quantitative RT-PCR (qRT-PCR)

Total RNA was extracted from kidney tissue or cells using Trizol (Invitrogen, Carlsbad, CA, USA). RNA reverse transcription was performed using a PrimeScript RT reagent kit 127 (Takara, Otsu, Japan) according to the manufacturer's instructions. Gene transcripts were quantified using a SYBR Premix Ex Taq TM II kit (Takara, Otsu, Japan). GAPDH was used as an internal control. The expression levels relative to β-actin were calculated using the 2^−ΔΔCT^ method. Real-time primers used in this study are described in Table 1.

**Table 1:** Primers for real-time PCR analysis

Gene	Primer sequence (5′-3′)
GAPDH Fwd	ATCATCCCTGCATCCACT
GAPDH Rew	ATCCACGACGGACACATT
TNFα Fwd	ACGGCATGGATCTCAAAGAC
TNFα Rew	AGATAGCAAATCGGCTGACG
IL-23 Fwd	CAGCAGCTCTCTCGGAATCT
IL-23 Rew	TGGATACGGGGCACATTATT
CCL-3 Fwd	GCCCTTGCTGTTCTTCTCTG
CCL-3 Rew	GATGAATTGGCGTGGAATCT
iNOS Fwd	GGAATCTTGGAGCGAGTTGT
iNOS Rew	GCAGCCTCTTGTCTTTGACC
Ariginase Fwd	GCAGAGGTCCAGAAGAATGG
Ariginase Rew	AGCATCCACCCAAATGACAC
CX3CR1 Fwd	TCCCTTCCCATCTGCTCAG
CX3CR1 Rew	ACAATGTCGCCCAAATAACAGG
IL-4 Fwd	TCTGTAGGGCTTCCAAGGTG
IL-4 Rew	ATCGAAAAGCCCGAAAGAGT
MRC Fwd	GGAGGCTGATTACGAGCAGT
MRC Rew	CATAGGAAACGGGAGAACCA
IL-10 Fwd	CCAAGCCTTATCGGAAATGA
IL-10 Rew	TCCTGAGGGTCTTCAGCTTC
Ym1 Fwd	TTCTTGTCACAGGTCTGG
Ym1 Rew	TCCTTAGCCCAACTGGTATAG
TLR4 Fwd	CCTGATGACATTCCTTCT
TLR4 Rew	AGCCACCAGATTCTCTAA
Myd88 Fwd	GCCAGAGTGGAAAGCAGTGT
Myd88 Rew	TATCGTTGGGGCAGTAGCAG
P65 Fwd	TAACAGCAGGACCCAAGGAC
P65 Rew	AGCCCCTAATACACGCCTCT

### Western blotting analysis

Harvested cells or collected tissues were lysed in radioimmunoprecipitation assay (RIPA) buffer (Beyotime Biotechnology, Shanghai, China) with protease inhibitor (Sigma-Aldrich, Darmstadt, Germany). The protein was quantified using the bicinchoninic acid (BCA) protein assay (Thermo Scientific, Waltham, MA, USA). Total proteins were resolved by 12% Tris-glycine sodium dodecyl sulfate polyacrylamide gel electrophoresis and were transferred to a polyvinylidene fluoride membrane (Millipore, Darmstadt, Germany). Membranes were blocked with 5% nonfat dry milk in PBST for 1 h and then incubated with primary antibodies against mouse Fibronectin (ab45688, Abcam), TLR4 (ab13867, Abcam), NF-κB (#8242, CST), phospho-NF-κB p65 (#3031, CST), MyD88 (#4283, CST) or GAPDH (#5174, CST) followed by incubation with a peroxidase-conjugated goat anti-rabbit (or mouse) IgG antibody. Densitometry analysis was performed, and the results were normalized to GAPDH expression and expressed as the fold changes over controls.

### Data analysis

All statistical analyses were performed using SPSS (version 19.0.0, SPSS Inc., Chicago, IL, USA). The results are expressed as the means ± standard deviations. Differences between groups were analyzed using two-sided Student's *t*-test or one-way ANOVA. Statistical significance was set at *P* < 0.05.
